# Optimizing enzyme thermostability by combining multiple mutations using protein language model

**DOI:** 10.1002/mlf2.12151

**Published:** 2024-12-26

**Authors:** Jiahao Bian, Pan Tan, Ting Nie, Liang Hong, Guang‐Yu Yang

**Affiliations:** ^1^ State Key Laboratory of Microbial Metabolism, Joint International Research Laboratory of Metabolic & Developmental Sciences, School of Life Sciences and Biotechnology Shanghai Jiao Tong University Shanghai China; ^2^ Institute of Key Biological Raw Material Shanghai Academy of Experimental Medicine Shanghai China; ^3^ Shanghai Artificial Intelligence Laboratory Shanghai China; ^4^ Shanghai National Center for Applied Mathematics (SJTU Center) & Institute of Natural Sciences Shanghai Jiao Tong University Shanghai China

**Keywords:** combinatorial mutants, creatinase, epistasis, protein language model, thermostability

## Abstract

Optimizing enzyme thermostability is essential for advancements in protein science and industrial applications. Currently, (semi‐)rational design and random mutagenesis methods can accurately identify single‐point mutations that enhance enzyme thermostability. However, complex epistatic interactions often arise when multiple mutation sites are combined, leading to the complete inactivation of combinatorial mutants. As a result, constructing an optimized enzyme often requires repeated rounds of design to incrementally incorporate single mutation sites, which is highly time‐consuming. In this study, we developed an AI‐aided strategy for enzyme thermostability engineering that efficiently facilitates the recombination of beneficial single‐point mutations. We utilized thermostability data from creatinase, including 18 single‐point mutants, 22 double‐point mutants, 21 triple‐point mutants, and 12 quadruple‐point mutants. Using these data as inputs, we used a temperature‐guided protein language model, Pro‐PRIME, to learn epistatic features and design combinatorial mutants. After two rounds of design, we obtained 50 combinatorial mutants with superior thermostability, achieving a success rate of 100%. The best mutant, 13M4, contained 13 mutation sites and maintained nearly full catalytic activity compared to the wild‐type. It showed a 10.19°C increase in the melting temperature and an ~655‐fold increase in the half‐life at 58°C. Additionally, the model successfully captured epistasis in high‐order combinatorial mutants, including sign epistasis (K351E) and synergistic epistasis (D17V/I149V). We elucidated the mechanism of long‐range epistasis in detail using a dynamics cross‐correlation matrix method. Our work provides an efficient framework for designing enzyme thermostability and studying high‐order epistatic effects in protein‐directed evolution.

## Impact statement

In this study, we developed a novel strategy for combining multiple positive mutations using a deep learning‐based protein language model. Our fine‐tuned AI model effectively captured complex long‐range epistasis and accurately predicted the optimal combination of multiple beneficial mutations. By using this approach, we successfully combined 18 single‐point mutants in just two rounds of design, achieving a 100% success rate. Traditionally, this would require 18 rounds of directed evolution. Additionally, the dynamics cross‐correlation matrix analysis elucidated the mechanism of dynamically mediated long‐range epistasis, enhancing our understanding of the impacts of mutations during the evolutionary process.

## INTRODUCTION

Protein engineering to enhance enzyme thermostability is crucial for expanding the applications of natural enzymes in industrial settings^1^, ^2^, ^3^. Over the past 20 years, (semi‐)rational design and random mutagenesis strategies have proven effective in identifying single‐point mutants that improve enzyme stability^4^, ^5^, ^6^, ^7^. However, combining multiple mutation sites to create optimized variants often introduces unwanted epistasis in the vast combinatorial sequence space, which can lead to the complete inactivation of the combinatorial mutants^8^, ^9^, ^10^. Epistasis indicates that residues within a protein interact with each other, so the effects of multiple mutations are not simply the sum of the effects of individual single‐point mutations^11^. This complexity means that combining multiple mutations often requires numerous rounds of trial and error, making the process highly time‐consuming. A recent approach, the stepwise combination method based on a greedy strategy, also depends heavily on multiple rounds of repetitive experiments^12^, ^13^, ^14^. This approach has the disadvantage of a significant waiting time for the results of previous rounds, severely limiting the efficiency of protein engineering.

Complex epistasis between mutations, which refers to the nonadditivity of the effects of two or more amino acids, presents a major obstacle to accurately predicting the optimal combination of mutations, particularly for high‐order combinatorial mutants involving three or more mutations^15^, ^16^, ^17^, ^18^, ^19^. Research on both natural and laboratory protein evolution has shown that epistasis is more common than previously recognized^20^. Notably, some functional mutations that are neutral or detrimental in the wild‐type (WT) background may become beneficial at later stages of evolution, indicating that these mutations are unpredictable from the starting point^21^. Capturing the epistasis between mutations accurately remains a challenge in efficiently exploring the combinatorial sequence space^18^. Addressing this challenge could significantly reduce combinatorial complexity and improve the efficiency of enzyme engineering.

The rapid advancement of large language models (LLMs) has sparked a revolution in artificial intelligence. Recently, several large‐scale protein language models (PLMs) have been introduced, including the ESM model family^22^, ^23^, ProtTrans^24^, ProGen^25^, and xTrimoPGLM^26^. These models are trained on extensive protein data sets containing tens of millions of sequences, allowing them to capture evolutionary patterns and sequence features in vector space^24^, ^26^, ^27^. However, these models are typically trained on a vast number of unlabeled sequences from databases like UniProt^28^ and Pfam^29^. As a result, the learned representations primarily capture the general context of broad protein families and do not provide detailed interpretations of specific protein properties, such as thermostability and enzyme activity. Consequently, most protein language models have not fully leveraged experimentally measured data with labels, leaving room for improvement in the accuracy and efficiency of functional predictions^30^, ^31^, ^32^.

We developed Pro‐PRIME, protein language model for intelligent masked pretraining and environment (temperature) prediction, based on a data set of optimal growth temperatures (OGTs) from 96 million host bacterial strains^33^. Pro‐PRIME uses a multi‐task learning paradigm to capture temperature‐related features associated with sequences in the data set. This approach enables Pro‐PRIME to assign higher scores to sequences that show enhanced temperature tolerance and align with natural biological principles. Consequently, Pro‐PRIME excels in designing and optimizing enzymes that require high‐temperature tolerance. Furthermore, by fine‐tuning the pre‐trained weights and incorporating a limited number of experimental results to adjust the parameters of the protein language models, we can significantly enhance predictive accuracy. This is particularly effective for predicting high‐order epistasis, which involves interactions between three or more mutations. Therefore, we plan to integrate Pro‐PRIME with supervised tuning, as this strategy could effectively address the challenge of efficiently combining multiple mutations.

In this study, we selected creatinase, a hydrolase crucial for diagnosing human kidney function^34^, to assess the feasibility of our strategy for practical thermostability engineering. In previous research, we aimed to enhance the stability of creatinase from *Alcaligenes faecalis* by designing and screening approximately 20 significant single‐point mutants using a non‐biased phylogenetic consensus method^35^. However, evaluation of the thermostability of each mutant required individual purification and characterization, consuming significant time and resources. Despite applying a stepwise combination strategy, we were only able to obtain a limited number of low‐order combinatorial mutants, including double‐, triple‐, and quadruple‐point mutants. This approach explored only a small portion of the entire combinatorial sequence space within the available time frame. In this study, we introduced a novel approach for enhancing enzyme thermostability by efficiently recombining beneficial single‐point mutants with the help of a protein language model. We fine‐tuned the Pro‐PRIME model using thermostability data from multiple single‐point mutants and some low‐order combinatorial mutants of creatinase. This allowed us to accurately predict changes in protein stability and identify optimal combinations of single‐point mutants. Additionally, we used a dynamic correlation matrix based on the three‐dimensional structure to conduct a mechanistic analysis of the complex epistasis observed in high‐order combinatorial mutants identified by the Pro‐PRIME model. This provides a deeper understanding of how mutations influence thermostability.

## RESULTS

### Efficient combination of multiple mutations guided by the fine‐tuned model

In our previous study^35^, we developed an unbiased consensus method to enhance the thermostability of creatinase. By analyzing the frequency of amino acid residues in the phylogenetic tree, we identified numerous candidate residues and screened them to obtain 18 single‐point mutants with increased half‐life (*t*_1/2_). We then combined these positive mutations using a stepwise combinatorial strategy, resulting in several mutants with further improved thermostability. This approach yielded 22 double‐point mutants, 21 triple‐point mutants, and 12 quadruple‐point mutants. To enable consistent comparison of their thermostability and catalytic activity, we measured the melting temperature (*T*_m_) values and relative activities of these mutants, which were used as the initial data set for this work (Table S1). Among the 18 single‐point mutants, four had lower *T*_m_ values compared to the WT: G58D, N130S, I240K, and K351E. In contrast, four mutants showed significantly higher *T*_m_ values than WT (Δ*T*_m_ > 1°C): D17V, V174I, T199S, and T251C. Most single‐point mutants were located on the surface of the creatine kinase structure (Figure S1).

**Figure 1:**
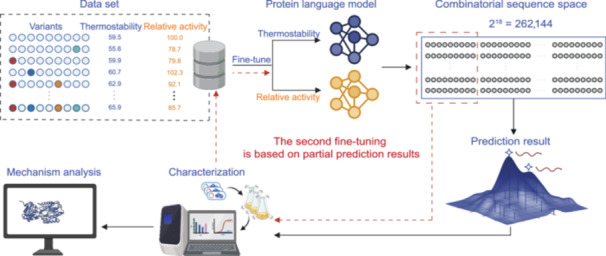
Flowchart of the strategy for combining stable mutations based on a pre‐trained model. The overall process includes four steps: (1) initial data collection; (2) fine‐tuning of the protein language model; (3) prediction of all mutants in the combined sequence space; and (4) validation of the selected mutants. The red dashed line shows the second round of model fine‐tuning via additional data from mutants predicted in the first round.

We aimed to develop a model that rapidly predicts changes in protein stability by combining pre‐trained molecular environment representations with supervised fine‐tuning. This approach would guide the design of combinatorial mutants with enhanced thermostability. Figure 1 illustrates the overall strategy. First, we fine‐tuned the Pro‐PRIME model to create a regression model for thermostability and a discriminant model for relative activity, using the initial data set with *T*_m_ and relative activity as labeled values, respectively. Next, we used the fine‐tuned Pro‐PRIME models to predict the thermostability and activity of all mutants within the combinatorial sequence space.

In enzyme engineering, there is often a trade‐off between activity and stability, meaning that optimizing both parameters simultaneously or equally is challenging^36^. Given our primary goal of enhancing the thermostability of creatinase, we aimed to avoid missing mutants that might slightly reduce activity but significantly improve stability. Therefore, we considered mutants with more than 60% relative activity compared to the WT as acceptable. Finally, we characterized the selected mutants to obtain experimental data and provided these data as feedback to further fine‐tune the model. The Pro‐PRIME model, developed from previous research, was trained on a data set containing the OGT of 96 million sequence host bacterial strains. It demonstrated superior predictive performance compared to other state‐of‐the‐art models when validated across multiple data sets and instances.

Using the fine‐tuned Pro‐PRIME model, we predicted the stability and activity distribution of all combinatorial mutants derived from the 18 single‐point mutants (2^18^ = 262,144). As the initial data set included a maximum of quadruple‐point mutants, we extended the validation to mutations involving 5, 6, 7, 8, and 9 points to assess the model's ability to predict the performance of high‐order combinatorial mutants based on low‐order mutation data. Our primary criterion for screening was the ranking of predicted thermostability scores. As a result, we selected a total of 25 mutants (Table S2), representing the top performers among variants with 5, 6, 7, 8, and 9 mutations.

**Figure 2:**
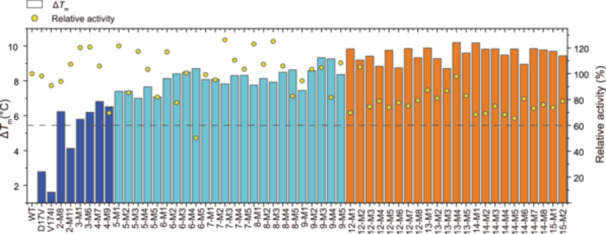
Thermostability and relative activity data for the designed mutants. Yellow circles show the relative activity. Blue bars show thermostability data for some mutants in the initial data set. Cyan and orange bars show thermostability data for all mutants in the first‐ and second‐round prediction, respectively. Relative activity is quantified by comparing the activity of the mutants to that of the wild‐type by using a coupled enzymatic assay system. Thermostability is quantified using the differential scanning fluorimetry (DSF) method, which allows for high‐throughput measurement of the *T*_m_ of mutants.

The results from the first round of predictions using the fine‐tuned model demonstrated excellent performance. We successfully improved the thermostability of all 25 mutants designed in this round compared to the WT, with increases ranging from 7°C to 9.33°C. Furthermore, the activity level of most mutants remained above 80% of that of the WT enzyme (Figure 2). Notably, the *T*_m_ values of these 25 mutants surpassed those of all mutants in the initial data set, indicating a 100% success rate for the model in designing stable mutants.

To enhance the prediction accuracy further, we integrated the characterization results into the data set and conducted a second round of fine‐tuning, prediction, and selection (Figures 1 and 2). In the second round, we selected the top 25 ranked mutants based on their thermostability prediction scores within the combinatorial sequence space. These mutants contained 12–15 mutations (Table S3).

**Figure 3:**
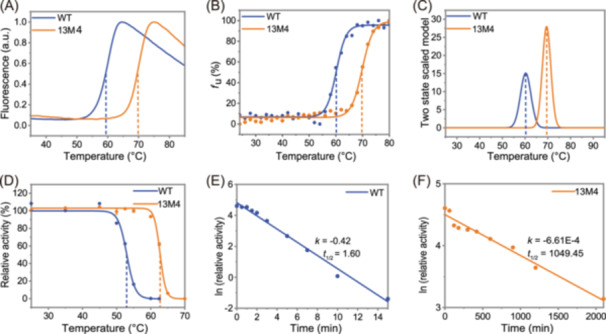
Thermodynamic and kinetic stability of WT and 13M4. Thermodynamic stability parameters include *T*_m_ and enthalpy change (Δ*H*). The *T*_m_ values for both wild‐type (WT) and 13M4 were quantified by DSF (A), CD spectra (B), and DSC (C). In addition, Δ*H* could be calculated from the results of DSC (C). The dashed lines correspond to the *T*_m_ of creatinase. Kinetic stability parameters include *T*_50_^15^ and *t*_1/2_. *T*_50_^15^ was derived from the results of the thermal inactivation curves of WT and 13M4 (D), as indicated by the dashed lines. The *t*_1/2_ was calculated from the thermal inactivation curve of WT (E) and 13M4 (F) under heat treatment at 58°C. CD, circular dichroism; DSC, differential scanning calorimetry; *f*_u_, unfolding fraction; *k*, the observed first‐order deactivation rate constant.

Compared to the WT, the thermostability of the 25 mutants predicted in the second round was further improved, with increases ranging from 8.17°C to 10.19°C, and overall activity was maintained above 60% (Figure 2). The best‐performing mutant in this round, 13M4, had 13 mutations and showed a *T*_m_ increase of 10.19°C, with activity comparable to the WT (Figure 3A and Table S3). This round showed minimal differences between mutants, with a slight overall decrease in activity. Therefore, we decided not to pursue further iterations of the predictive model.

### Thermodynamic and kinetic stability of the best‐stabilized variant

To further validate the enhanced thermostability of the composite mutants identified during the screening process, we conducted a comprehensive characterization of the thermodynamic and kinetic stability of the most favorable mutant, 13M4, in comparison to the WT, as shown in Figure 3. The *T*_m_ of proteins is the temperature at which 50% of the protein molecules unfold. It reflects the changes in protein conformation with temperature and serves as a key indicator of thermodynamic stability. We used three commonly used methods to determine protein *T*_m_ values: DSF, differential scanning calorimetry (DSC), and circular dichroism (CD) spectroscopy. Each technique measures different aspects of protein behavior during heating. DSF detects the exposure of hydrophobic groups within proteins as they unfold with heating. DSC assesses thermodynamic parameters by measuring the difference in power required to maintain the sample and reference at the same temperature. CD spectroscopy evaluates changes in the secondary structure of proteins throughout the heating process^37^.

The outcomes from the different methods were consistent, as shown in Figure 3A–C. Compared to the WT, the 13M4 mutant showed a significantly higher *T*_m_, with an approximate increase of 10°C (ranging from 9.24°C to 10.19°C). We also used the DSC method to measure the enthalpy change (Δ*H*), which quantifies the energy required for the protein unfolding reaction^38^. The Δ*H* of 13M4 was approximately 200 kJ/mol higher than that of WT, indicating stronger internal attractive forces in 13M4. These results highlight the significant enhancement in the thermodynamic stability of 13M4.

For kinetic stability, we assessed the semi‐inactivation temperature (*T*_50_^15^) and half‐life (*t*_1/2_) of both WT and 13M4. WT became completely inactive after 15 min of incubation at 60°C, while 13M4 retained 93.5% of its activity under the same conditions. Additionally, 13M4 showed a notable increase in *T*_50_^15^, being 10°C higher than that of WT (Figure 3D). At 58°C, the *t*_1/2_ of WT was only 1.6 min (Figure 3E), whereas 13M4's *t*_1/2_ extended to 1049.45 min (Figure 3F), representing a remarkable 655.91‐fold enhancement.

In terms of catalytic efficiency, 13M4 maintained a level of activity (98%) comparable to that of the WT. Catalytic kinetic results showed that the *k*_cat_ values of 13M4 and WT were similar. However, the *K*_m_ value for 13M4, which indicates substrate affinity, was slightly improved (Figure S2). Detailed data on stability and kinetic parameters are provided in Table 1. These results demonstrate that 13M4 is significantly more thermally stable than WT and holds promising potential for industrial applications.

**Table 1:** Thermodynamic and kinetic parameters of wild‐type (WT) and 13M4.

	WT	13M4
*T*_m__DSF (°C)	59.53 ± 0.33	69.72 ± 0.15
*T*_m__CD (°C)	60.18 ± 0.22	69.84 ± 0.23
*T*_m__DSC (°C)	60.29	69.53
Δ*H* (kJ/mol)	614.5	837.2
*T*_50_^15^ (°C)	52.86 ± 0.29	62.90 ± 0.12
*t*_1/2__58°C (min)	1.60 ± 0.01	1049.45 ± 38.33
Relative activity (%)	100 ± 5.19	97.99 ± 1.58
*k*_cat__37°C (s^−1^)	28.72 ± 1.42	26.04 ± 0.82
*K*_m__37°C (mM)	10.46 ± 1.72	6.21 ± 0.77

### Molecular mechanisms underlying higher thermostability of 13M4

After thoroughly characterizing the optimal mutant and the WT, we aimed to investigate the molecular basis for the observed improvements in thermostability through structural analysis and molecular dynamics (MD) simulations. We used the WT structure obtained from our previous study, where we elucidated the crystal structure of WT using X‐ray crystallography (PDB 7YTO)^39^. We modeled the structure of mutant 13M4 based on the WT crystal structure coordinates and used MD simulations to equilibrate and refine the in silico mutant model (Figure S3 shows the structural comparison before and after mutation in 13M4). We conducted 100 ns MD simulations of both WT and 13M4 at 62°C. The detailed simulation protocol is provided in the Materials and Methods section.

**Figure 4:**
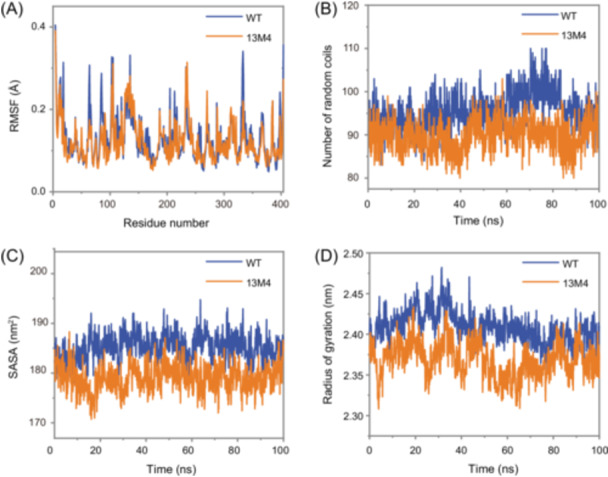
Results of the molecular dynamics simulation before and after mutation. Root mean square fluctuation (RMSF) (A), number of random coils (B), solvent‐accessible surface area (SASA) (C), and radius of gyration (Rg) (D) of WT and 13M4 were obtained from molecular dynamics simulations.

Analyses of root mean square fluctuation (RMSF), number of random coils, solvent‐accessible surface area (SASA), and radius of gyration (Rg) revealed significant rigidification and compaction of the 13M4 mutant (Figure 4). Specifically, the RMSF of 13M4 was lower than that of the WT, indicating that the atoms or residues in 13M4 experienced less displacement during the simulation and were more stable compared to WT (Figure 4A). The average number of random coils was also lower in 13M4 than in WT, likely because the mutations in 13M4 reduced the number of unstructured regions, decreasing flexibility and increasing rigidity between molecules (Figure 4B). Additionally, the average SASA of 13M4 was lower than that of WT, suggesting that a greater proportion of 13M4's surface was covered by atoms within the molecule, leading to a reduced contact area with the solvent (Figure 4C). SASA is a crucial factor in protein folding and stability studies. Generally, in protein engineering, lower SASA values indicate a more compact and stable structure for mutant enzymes^40^, ^41^. The lower SASA observed in mutant 13M4 compared to the WT indicated a tighter packing of its structure. This tighter packing made 13M4 less prone to unfolding at high temperatures and reduced the impact of solvent molecules on its internal structure, thereby enhancing overall enzyme stability. The Rg of 13M4 was also lower than that of WT, suggesting that 13M4 is smaller and more compact. Rg measures the compactness of a molecule's overall shape, and smaller Rg values typically indicate a more compact structure (Figure 4D). These quantitative results suggest that the mutations in 13M4 have increased the rigidity of the enzyme by reducing local dynamics and unfolding motions while compressing the tertiary structure to limit exposure. This finding is consistent with the observed increase in Δ*H*. Thus, the combined effects of reduced flexibility and enhanced compactness likely contribute significantly to the enzyme's resistance to thermal denaturation, providing substantial thermal stabilization.

**Figure 5:**
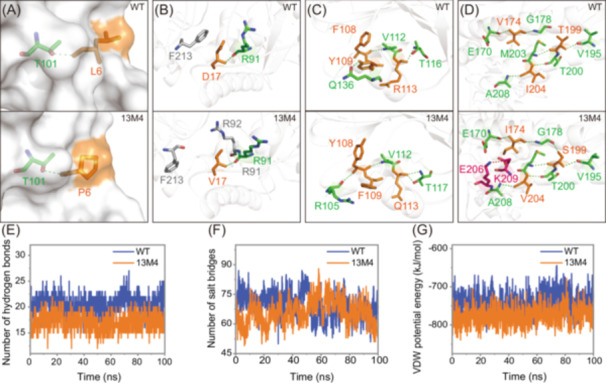
Structural comparisons between WT and 13M4 near the mutations. (A–D) The inter‐residue interactions around the mutations: L6P, D17V, F108Y/Y109F/R113Q, and V174I/T199S/I204V, respectively. The mutated amino acids are shown in orange. Residues involved in hydrogen bonding, salt bridges, and van der Waals interactions are shown in green, purple, and gray, respectively. Hydrogen bonds and salt bridges are shown with green and purple dashed lines. Molecular dynamics simulations were used to calculate the number of hydrogen bonds (E), salt bridges (F), and van der Waals force energy (G) of the creatinase before and after the mutation.

We further explored the molecular basis of the enhanced thermostability by analyzing the modeled structure of mutant 13M4. Figures 5 and S4 show the structural comparison between the WT and 13M4 in the vicinity of the mutations. We examined changes in inter‐residue interactions around the mutation sites. The single‐point mutation (L6P) introduced a proline substitution in a flexible region, reducing the conformational entropy of the locally unfolded protein and resulting in a more rigid spatial structure (Figure 5A). Similar patterns of increased rigidity have been observed in other modified enzyme molecules^4^, ^42^. Specifically, the D17V mutation introduced three additional van der Waals interactions (Figure 5B). The F108Y/Y109F/R113Q mutation caused a significant rearrangement of the hydrogen‐bonding network, with the R113Q variant adding one hydrogen bond (Figure 5C). The hydrogen‐bonding network was also rearranged around several other mutations, including I149V (Figure S4A), V174I/T199S/I204V (Figure 5D), and K351E (Figure S4C). Notably, the V174I/T199S/I204V mutation introduced an additional salt bridge, while the Q165I mutation resulted in a reduced hydrogen bond (Figure S4B). No direct interaction changes were observed around the T251C and E349V mutations (Figure S4C).

These findings indicate that multiple mutations significantly impact the interaction network throughout the protein, collectively enhancing stability. MD simulations quantified the changes in interaction forces before and after the mutations. The results showed that the number of hydrogen bonds in 13M4 decreased compared to WT (Figure 5E), while the number of salt bridges remained largely unchanged (Figure 5F). Conversely, van der Waals potential energy was decreased in 13M4 (Figure 5G).

### Epistatic effects of mutations captured by the fine‐tuned Pro‐PRIME model

**Figure 6:**
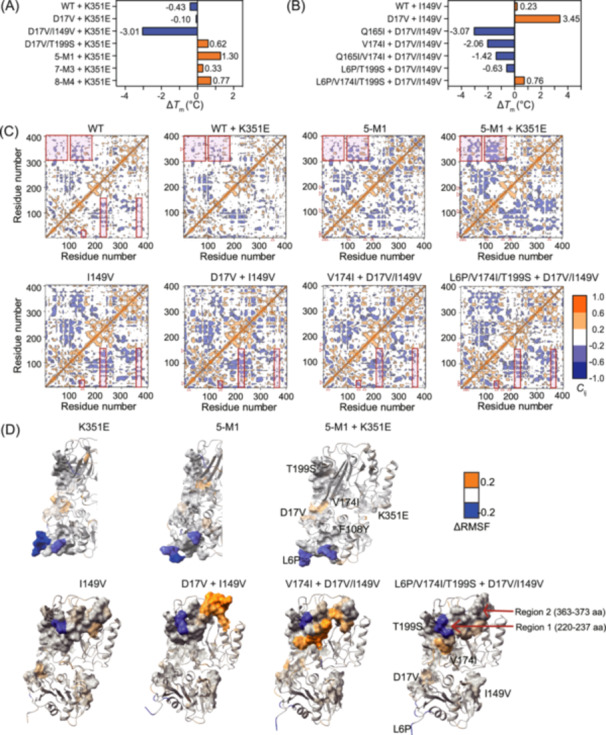
Analysis of epistatic effects between mutations. (A, B) Epistatic effects of K351E (A) and D17V/I149V (B) in *T*_m_ values. Blue indicates a negative effect and orange indicates a positive effect. The mutants analyzed include 5‐M1 (L6P/D17V/F108Y/V174I/T199S), 7‐M3 (L6P/D17V/F108Y/I149V/V174I/T199S/T251C), and 8‐M4 (L6P/D17V/F108Y/I149V/V174I/T199S/I204V/T251C). (C) Dynamics cross‐correlation matrix map for *C*_α_ atom pairs within monomers of creatinase WT and stable variants. The correlation coefficient (*C*_ij_) is depicted in various colors. Mutated sites are delineated with red arrows, and regions of significant kinetic correlation around mutations are highlighted in red boxes. (D) Variant structures colored by normalized RMSF change when compared to WT. The residues within a 5 Å radius around the mutation site (or associated region) are depicted on the surface.

The functional impact of an amino acid mutation often depends on the background sequence into which it is introduced. This dependence on the genetic background, known as epistasis^43^, ^44^, can significantly influence protein evolution. A mutation that is beneficial in one context may be deleterious or neutral in another^21^. Epistasis can occur either through direct interactions between amino acids or through distal, indirect interactions, with the potential for exponential increases in epistatic effects as the number of mutations increases^44^. Although capturing nonlinear and long‐range epistatic effects is challenging, our results indicate that the fine‐tuned Pro‐PRIME model successfully captures various epistatic interactions. For example, the K351E mutation demonstrated divergent effects depending on the mutational background, a phenomenon known as sign epistasis^19^. This mutation showed both positive and negative influences, as illustrated in Figure 6A. Another notable case involves the single‐point mutations D17V and I149V. These mutations displayed synergistic epistasis^19^, as shown in Figure 6B. The combination of D17V and I149V created a local optimum for protein thermostability. Introducing one or two additional beneficial mutations in this context reduced the *T*_m_ value, indicating that D17V/I149V established a stable intermediate. However, the introduction of three or more mutations, such as L6P/V174I/T199S, overcame this evolutionary barrier and further increased the *T*_m_ value (Figure 6B).

It is noteworthy that after two rounds of refinement, the Pro‐PRIME model effectively captured the epistatic effects present in the data set, which facilitated predictions of optimal combinations. The frequency of the K351E mutation increased significantly throughout the evolution process, with ratios of 4/73, 3/25, and 24/25 in the initial data set, and the first and second rounds of prediction results, respectively (Tables S1‐S3). This indicates that effective epistasis identified in low‐order mutants was consistently captured by the large protein language model, as evidenced by the increased likelihood of this mutation appearing through the fine‐tuning process. Similarly, the frequency of the D17V/I149V mutation increased steadily across the three data sets, reaching 14/73, 10/25, and 25/25, respectively (Tables S1‐S3). In contrast, the K351E mutation, which showed negative effects in the WT background during the stepwise combinatorial strategy, was either discarded or not prioritized in subsequent predictions.

These mutations displayed significant spatial separation (>25 Å) (Figure S5), making it difficult to analyze the epistatic mechanisms through local static structures. To explore the long‐range epistatic effects between mutations, we computed dynamics cross‐correlation matrices (DCCMs) for the WT and selected mutant variants. DCCMs illustrate the correlation coefficient (*C*_ij_) between specific atoms of each amino acid within a protein, such as *C*_α_ atoms, providing insights into the correlated motions of the protein^45^. *C*_ij_ values range from 0 to 1, indicating positive correlations, and from −1 to 0, indicating negative correlations. Positive correlations reflect simultaneous increases or decreases in local kinetics across both regions, while negative correlations indicate opposing changes.

Our analyses revealed that mutations altered the negative correlations among regions of interest, particularly those adjacent to the mutation sites. These regions are highlighted by red boxes in Figure 6C. These dynamics‐related changes mediated the observed long‐range epistasis. For example, the introduction of the K351E mutation into the WT background diminished the negative correlation between the region near position 351 and the regions 1–90 amino acids (or 100–175 amino acids) (Figure 6C). This observation indicated that the K351E mutation increased the RMSF in its vicinity while also elevating RMSF in the regions 1–90 amino acids (or 100–175 amino acids) (Figure 6D).

Increased RMSF indicated greater instability in these regions, which explains the negative impact of the K351E single mutation on the *T*_m_ values of the WT (Figure 6A). However, when combined with higher‐order mutants (5‐M1, L6P/D17V/F108Y/V174I/T199S), the K351E mutation significantly enhanced the negative correlation between these regions (Figure 6C). This indicated that, in this context, the K351E mutation actually improved the local stability of regions 1–90 amino acids (or 100–175 amino acids) (ΔRMSF ≤ 0) (Figure 6D), thereby positively affecting the overall protein *T*_m_ value (Figure 6A).

Similarly, changes in dynamic correlations between D17V/I149V and other mutations aligned with the observed epistasis on *T*_m_ values when they were combined. For instance, in the triple‐point mutant D17V/I149V/V174I, we observed a reduced negative correlation between D17V/I149V and regions 1 (220–237 aa) and 2 (363–373 aa) (Figure 6C). Concurrently, D17V and I149V decreased their local stability (ΔRMSF > 0), which led to a reduction in the local stability of regions 1 and 2 (Figure 6D) and consequently a decrease in the mutant's *T*_m_ (Figure 6B). In the quintuple‐point mutant L6P/D17V/I149V/V174I/T199S, the negative correlation between D17V/I149V and regions 1 and 2 increased (Figure 6C). Although D17V and I149V were unfavorable for their own local stability, they improved the local stability of regions 1 and 2 (ΔRMSF ≤ 0) (Figure 6D), which explains the observed increase in *T*_m_ with the addition of D17V/I149V. These results demonstrate that mutations can affect not only their own local stability but also potentially influence the stability of other regions through interactions, resulting in non‐additive effects on stability known as epistasis. Such inter‐mutational interactions at the dynamic level can be partially detected and analyzed through dynamic correlation matrices.

## DISCUSSION

In this study, we successfully integrated deep learning with protein engineering to efficiently combine single‐point mutations. We used the Pro‐PRIME model, which we fine‐tuned based on a comprehensive data set of 99 experimental data points (74 mutations from the initial data set and 25 mutations from the first round of prediction). This approach allowed us to rapidly explore over 260,000 possible mutations (2^18^) within the combinatorial library. All 50 mutants that entered the experimental screening phase demonstrated improved thermostability, achieving a 100% success rate. We accurately combined 13 mutations in only two rounds of experiments. The best mutant, 13M4, showed a significant increase of 10.19°C in apparent melting temperature compared to the WT, indicating exceptional long‐term stability. Furthermore, 13M4 showed a 655.91‐fold improvement in the half‐life at 58°C while maintaining catalytic activity comparable to that of the WT. This makes 13M4 a promising candidate for creatinine detection applications. These results highlight the effectiveness of our customized design strategy in enhancing thermostability and provide a novel framework for improving the thermostability of other commercially important enzymes.

In the vast protein sequence space, the number of possible mutants is enormous, while functional proteins are relatively rare^9^. In recent years, researchers have developed various focused mutagenesis methods that require minimal screening. These methods include the combinatorial active‐site saturation test (CAST), iterative saturation mutagenesis (ISM), and rational enzyme design approaches such as focused rational iterative site‐specific mutagenesis (FRISM). These techniques enhance the efficiency of identifying positive mutants for attributes such as selectivity, activity, and thermostability^46^, ^47^. Despite these advances, effectively combining multiple positive mutants remains challenging. Traditional directed recombination or random mutation strategies typically require a large number of experiments^48^, ^49^, ^50^. Recently, the greedy strategy has become a common approach for combining multiple mutations. This strategy involves constructing and screening high‐order combinatorial mutants gradually by combining advantageous single‐point mutations based on existing best‐performing variants. However, when mutations interact epistatically, this combinatorial process may not easily identify effective evolutionary pathways. To address this issue, researchers have proposed improved greedy strategies^14^, ^51^. These strategies categorize mutations into different classes based on calculated parameters and then combine them stepwise to explore as many possibilities as possible. However, this approach still faces time constraints due to multiple rounds of combination. For example, Wu's group obtained the best mutant after 13 rounds of combination, a process that could take 13 to 39 weeks under conventional experimental conditions^14^.

In our study, we demonstrate that fine‐tuning protein language models with experimental data offer a more efficient solution for exploring protein sequence space. This approach allows us to rapidly explore all possible combinations, effectively avoiding local optima caused by epistatic effects while minimizing the workload and time required for screening. By performing only two rounds of design and validation with 50 mutants, we quickly identified multiple high‐order combinatorial mutants with improved thermostability, pinpointing the optimal combination of 13 sites. Each learning and prediction round required only 1 week, with most of the time spent waiting for computations to complete. Notably, the number of combinatorial mutants increased exponentially with the number of single‐point mutations. Thus, selecting single‐point mutations carefully is crucial, as the number of these mutations determines the computational resources needed to predict all combinatorial mutants. With adequate computational resources, it is possible to include a few valuable negative mutations in the single‐point mutation library, such as those that reduce thermostability but increase activity. These negative mutations might yield surprising results in the final combination.

Interestingly, upon reviewing the experimental data, we observed complex high‐order epistatic effects among certain mutations, even when these mutations were spatially distant. Epistasis disrupts smooth, additive, and predictable mutational behavior, altering gradients in the fitness landscape. This alteration can make the path to a global optimum lengthy or introduce local optima, where traditional directed evolution methods may become trapped^52^. Currently available mutation data sets often describe epistasis at only a few positions, typically single‐ and double‐point mutants. This limitation makes it challenging to detect complex epistasis in high‐order combinatorial mutants in protein evolution and engineering studies. In contrast, our strategy efficiently captures and predicts epistasis from both trained sequence data and data used for fine‐tuning. The fine‐tuned model accurately applied these long‐range interactions between mutations to predict combinatorial mutants. Results from dynamic correlation matrix analysis showed that mutations affecting stability influence not only the dynamics of their local environment but also, in some cases, impact the dynamics of distant structural regions^53^, ^54^, ^55^. This mechanism mediates long‐range epistasis, suggesting that future protein engineering strategies could utilize changes in correlated dynamic networks for a deeper analysis of epistasis. This insight prompted us to integrate structural data into protein language models, potentially providing these models with more detailed information about interactions between amino acid residues. Several models have already combined unannotated sequence data with structural data to train protein language models, achieving good predictive accuracy^56^, ^57^.

Moreover, computing DCCMs of mutations and incorporating them into machine learning models present an intriguing application. This approach could provide unique data on epistatic effects. Compared to wet experiments, data from MD simulations are more time‐efficient and cost‐effective, thereby enhancing the efficiency of protein engineering. In fact, combining MD simulation data with machine learning to construct models has been successfully implemented in various fields, including protein sequence design^58^, protein conformational ensemble generation^59^, and enzyme function prediction^60^.

In conclusion, we present a framework for enhancing protein engineering efficiency by fine‐tuning a general temperature‐guided language model with a small amount of experimental data. This approach allows us to construct a model capable of accurately identifying sequence–thermostability relationships and complex epistasis, thereby guiding the efficient combination of multiple significant single mutations. This framework could be readily applied to other important enzymes. Additionally, the kinetic analysis of complex epistasis offers a unique perspective for future mechanistic studies.

## MATERIALS AND METHODS

### Materials

The creatinase gene (*BAA88830.1*) was derived from *Alcaligenes faecalis*. In this study, we used the I304L/F395V creatinase variant (afCR‐M0)^35^ as the parent template to construct additional variants, which we refer to as WT. We cloned the creatinase gene into the pET‐28a(+) vector. We then transferred the recombinant plasmid into *E. coli* DH5α and *E. coli* BL21(DE3) for molecular cloning and protein expression, respectively. We purchased PrimerSTAR, *Dpn*I, and other reagents for the molecular cloning experiments from TaKaRa Biotechnology Co., Ltd. We acquired Oxoid™ Tryptone and Oxoid™ Yeast Extract Powder from Thermo Fisher Scientific. Sodium chloride and nickel sulfate were purchased from Shanghai Titan Scientific Co., Ltd. and Shanghai Macklin Biochemical Co., Ltd., respectively. Agar, tris, imidazole, ethylenediaminetetraacetic acid (EDTA), phosphate‐buffered saline (PBS) powder, kanamycin, and isopropyl‐β‐d‐thiogalactopyranoside (IPTG) were products of Sangon Biotech. We obtained SYPRO® Orange Protein Gel Stain from Sigma Aldrich. All other chemicals were of analytical‐grade purity.

### Protein expression and purification

We initially grew the recombinant expression strain in 3 ml of 2×YT medium containing kanamycin (100 μg/ml) overnight at 37°C with orbital shaking at 220 rpm. We then used the starter culture to inoculate 250 ml of 2×YT medium supplemented with kanamycin (100 μg/ml) in a shake flask. We incubated this flask under the same conditions until the OD_600_ of the culture reached between 0.6 and 0.8. We induced protein expression by adding IPTG to a final concentration of 421 μM and continued incubating the culture at 20°C with agitation at 220 rpm for 16–18 h. We harvested the cells by centrifugation at 8000 rpm for 20 min at 4°C, washed them, and resuspended them in binding buffer (20 mM Tris–HCl, 200 mM NaCl, 20 mM imidazole, pH 8.0). We disrupted the cells using a high‐pressure homogenizer and centrifuged the resulting lysate at 12,000*g* for 60 min to remove cellular debris. We applied the clarified supernatant to a pre‐equilibrated Ni‐NTA column and eluted the bound proteins using an imidazole gradient (20–200 mM). We evaluated the purity of the eluted fractions by SDS‐PAGE. We pooled the fractions containing the desired proteins, desalted them by ultrafiltration, concentrated them, and stored them at −80°C in 1× PBS buffer (10 mmol/l phosphate buffer, 137 mmol/l NaCl, 2.7 mmol/l KCl, pH 7.4).

### Activity assay

We evaluated the catalytic activity of creatinase using a coupled enzymatic assay system that included sarcosine oxidase and horseradish peroxidase. We diluted the creatinase sample to a final concentration of 1 mg/ml in 1× PBS buffer. We measured enzyme activity by combining 50 μl of the diluted enzyme with 950 μl of a reaction mixture containing 0.5 mM creatine, 0.45 mM 4‐aminoantipyrine (4‐AA), 0.5 mM N‐ethyl‐N‐(2‐hydroxy‐3‐sulfopropyl)−3‐methylaniline (TOOS), and 1× PBS buffer. We conducted the assay at 37°C and monitored the rate of change in absorbance at 555 nm using a UV2550 spectrophotometer. We defined one unit of enzymatic activity as the amount of enzyme required to produce 1 μM of hydrogen peroxide per minute under the described assay conditions.

### Thermodynamic stability analysis

DSF detects changes in the tertiary structure of proteins by measuring the exposure of hydrophobic groups. We performed this method by mixing protein samples with 5× SYPRO Orange stain at a final concentration of 6 μM and placing them in octuplex PCR tubes. We standardized the volume of each sample to 20 μl and conducted three separate tests for each sample. We determined denaturation curves using appropriate filter pairs (FAM470nm for excitation and ROX625nm for emission) in a PCR instrument (Analytik Jena qTower3). We increased the temperature from 25°C to 95°C in 0.5°C increments, holding each temperature step for 5 s to allow equilibrium. We approximated the melting temperature of the thermal expansion curve by fitting the Boltzmann equation^61^, ^62^. We selected this method to determine the *T*_m_ values of the mutants listed in Tables S1–S3 for its high‐throughput advantage.

We performed nano differential scanning calorimetry (nanoDSC) analyses using nanoDSC equipment from TA Instruments. We prepared the samples by diluting them to a concentration of 1 mg/ml with 1× PBS buffer solution and then loaded them into high‐sensitivity capillaries for detection. During the experiment, we heated the proteins through a controlled thermal ramp, increasing the temperature from 20°C to 95°C at a constant rate of 1°C per minute to induce and monitor the unfolding transition. We subjected the protein unfolding process to this thermal ramp (20–95°C, 1°C/min). We analyzed the results, including *T*_m_ and Δ*H*, using Launch NanoAnalyze software with the TwoStateScaled model to fit the experimental data.

We used CD to detect thermal inactivation processes in proteins by measuring the decrease in polarized light absorption as the secondary structure is disrupted. We performed heat‐up experiments with a Jasco J‐1500 spectrometer using a 1 mm optical path cuvette. We monitored the far‐UV CD signal (216 nm) as the temperature increased from 25°C to 80°C at a heating rate of 1°C/min, recording data at 2°C intervals. We calculated the unfolding fraction (*f*_u_) using $f_{u}=\frac{{\mathrm{CD}}_{T}-{\mathrm{CD}}_{\min}}{{\mathrm{CD}}_{\max}-{\mathrm{CD}}_{\min}}\times 100\%$, where CD_T_ is the observed CD value at a specific temperature, and CD_max_ and CD_min_ are the maximum and minimum CD values, respectively, during the thermal inactivation process of the proteins.

### Kinetic stability analysis

To determine the kinetic stability of the enzyme, we diluted the purified enzyme to 1.0 mg/ml in 1× PBS buffer. We assessed the *t*_1/2_ of the enzyme by incubating it at 62°C for various time periods. We then measured the residual enzyme activity at 37°C using the previously described methodology. We calculated the enzyme's *t*_1/2_, which represents the time required for the enzyme's initial activity to decrease by half, using the first‐order deactivation function: *t*_1/2_ = −ln (2)/*k*, *k* * t = ln (Ar/A0), where *k* is the observed first‐order deactivation rate constant. Ar and A0 represent the residual activity and initial activity of the enzyme, respectively.

We determined the *T*_50_^15^ value by incubating the purified enzyme at temperatures ranging from 25°C to 70°C for 15 min. After incubation, we allowed the sample to cool in an ice bath. We then quantified the residual enzymatic activity using the previously outlined methodology. The *T*_50_^15^ value indicates the temperature at which the enzyme activity is reduced to 50% after 15 min of heat treatment, with the highest activity considered to be 100%. We analyzed the data by calculating the inflection point of the residual activity fitted to an S‐shaped plot using the Boltzmann function in Origin software at a specific temperature.

### Kinetic parameters

We measured the kinetic parameters (*K*_m_ and *k*_cat_) in 1× PBS buffer using various concentrations of the substrate creatine (0.5, 2, 5, 8, 10, 14, 18, 30, 50, 80, and 120 mM). We conducted the reactions as previously described. We determined the reaction kinetics parameters by applying the Michaelis–Menten model to the obtained data.

### Fine‐tuning of the Pro‐PRIME

For the training process, we converted each mutant sequence, labeled appropriately, into a latent space representation (L × 1280) using Pro‐PRIME's transformer‐encoder, where L represents the protein length. We averaged the 1280‐dimensional amino acid vectors to create a representative vector for each sequence. This representative vector served as input for a regression head layer that included an MLP layer, a dropout layer, and a Tanh activation function, yielding a single prediction score for each mutant. We used the Adam optimizer and MSE as our loss function with a maximum of 200 training epochs. We applied early termination if MSE did not improve over 10 epochs. All training was performed on a single 80GB NVIDIA A100 GPU card, with a learning rate of 1e−5 and a batch size of 4.

### Molecular dynamics simulations

We used the X‐ray crystal structure of WT creatinase from previous work (PDB 7YTO)^39^ as the starting structure for the MD simulation. For the 13M4 mutant, we used the same structure as WT but incorporated 13 residue mutations as described in the experiment. Our simulation box contained a single creatinase molecule, with a minimum distance of 1 nm between the protein and the box boundary. We added water molecules to the box and supplemented it with 0.1 mol/l NaCl to ensure charge neutrality. We simulated the protein using the CHARMM27 force field and the water using the TIP3P model. We conducted the simulations using GROMACS‐2021. We truncated the van der Waals interactions at 1.2 nm using the Lennard–Jones potential, which was gradually switched to zero at 1.0 nm. We calculated electrostatic interactions using Particle Mesh Ewald with a Coulomb cutoff of 1.2 nm. We specifically restrained hydrogen‐containing bonds using the LINCS method, which allowed for a simulation time step of 2 fs. We initially minimized the system's energy using the steepest descent techniques, with a threshold force of 10.0 kJ per mole per nanometer and up to 50,000 iterations.

We first equilibrated the system at a constant volume and temperature (NVT ensemble) at 335.15 K for 50 ns. We then continued equilibration at constant pressure and temperature (NPT ensemble) at 1 bar for an additional 50 ns. We regulated the temperature using the velocity‐rescale algorithm with a coupling period of 0.1 ps. We controlled the pressure using the Parrinello–Rahman method with a coupling constant of 3 ps. Next, we performed the production phase of the MD simulation under NPT conditions for 200 ns, maintaining a time step of 2 fs. For analysis, we used the final 100 ns of the simulation trajectories, recorded at 100‐ps intervals. We visualized the protein structures using the PyMOL package.

### Dynamics cross‐correlation matrix

We calculated the DCCMs according to the guidelines^63^. We determined the mutant's structure by modifying the corresponding amino acid in the WT crystal structure. We computed the DCCMs using Bio3D^64^, calculating the correlation coefficients for the *C*_α_ atoms. We archived the final 10 ns of the MD simulation trajectory at 10‐ps intervals and converted the data into the dcd file format using the CatDCD extension in VMD software^65^. We then input the resulting trajectory into Bio3D. We derived the correlation matrices from averaging across three independent simulation trajectories and graphically represented them using the Origin software package.

## AUTHOR CONTRIBUTIONS

**Jiahao Bian**: Data curation (equal); investigation (lead); validation (lead); writing—original draft (lead); and writing—review and editing (equal). **Pan Tan**: Data curation (equal); formal analysis (equal); and writing—review and editing (equal). **Ting Nie**: Data curation (equal); formal analysis (equal); and writing—review and editing (equal). **Liang Hong**: Conceptualization (equal); supervision (equal); and writing—review and editing (equal). **Guang‐Yu Yang**: Conceptualization (equal); funding acquisition (lead); supervision (equal); and writing—review and editing (equal).

## ETHICS STATEMENT

This study did not involve animals or humans.

## CONFLICT OF INTERESTS

The authors declare no conflict of interests.

## Supporting information

Supporting information.

## Data Availability

Benchmark data sets used for the Pro‐PRIME model are available within the article (https://doi.org/10.48550/arXiv.2307.12682) and on the website https://github.com/ai4protein/Prime). The data supporting the findings for creatinase are available within the article and its supplementary materials.
