# Supplementary material for: Optimizing enzyme thermostability by combining multiple mutations using protein language model
Source: mLife. 2024 Dec 26;3(4):492–504. doi: 10.1002/mlf2.12151 (PMC11685841; doi:10.1002/mlf2.12151)
Supplement: Supplementary file 1 — Supporting information. [file MLF2-3-492-s001.doc]

**Supplemental materials**

**Insight into High-order Epistasis and Efficiently Combining Multiple Mutations to Enhance Enzyme Thermostability Using Temperature-Guided Protein Language Model**

Jiahao Bian^1,2#^, Pan Tan^3,4#^, Ting Nie^1,2#^, Liang Hong^3,4^, Guang-Yu Yang^1,2*^.

1. *State Key Laboratory of Microbial Metabolism, Joint International Research Laboratory of Metabolic & Developmental Sciences, School of Life Sciences and Biotechnology, Shanghai Jiao Tong University, Shanghai 200240, China*
2. *Institute of Key Biological Raw Material, Shanghai Academy of Experimental Medicine, Shanghai 201401, China*
3. *Shanghai Artificial Intelligence Laboratory, Shanghai 200240, China*
4. *Shanghai National Center for Applied Mathematics (SJTU Center), & Institute of Natural Sciences, Shanghai Jiao Tong University*

# Contributed equally to this work.

*To whom correspondence should be addressed: [yanggy@sjtu.edu.cn](mailto:yanggy@sjtu.edu.cn)

**Contains 3 supplemental tables and 5 supplemental figures:**

Table S1. Tm values and relative activity data for mutants in the initial dataset. (Page 2-3)

Table S2. Tm values, relative activity and prediction data for mutants in the first round of prediction. (Page 4-5)

Table S3. Tm values, relative activity and prediction data for mutants in the second round of prediction. (Page 6-8)

Figure S1. Distribution of the single-point mutations in the creatinase structure. (Page 9)

Figure S2. Catalytic kinetics of WT and 13M4. (Page 10)

Figure S3. Distribution of the mutated amino acids in the structures of WT and 13M4. (Page 11)

Figure S4. Structural comparison between WT and 13M4 near the mutations. (Page 12)

Figure S5. Distances between the mutations with epistasis. (Page 13)

Table S1. T_m_ values and relative activity data for mutants in the initial dataset.

| ID | Mutation | Tm_DSF (℃) | Error of T_m_ (±) | Relative activity (%) | Error of relative activity (±) |
| --- | --- | --- | --- | --- | --- |
| WT | M0(I304L_F395V) | 59.534 | 0.33 | 100 | 5.19 |
| L6P | L6P | 59.881 | 0.255 | 86.697 | 5.838 |
| D17V | D17V | 62.324 | 0.306 | 98.165 | 5.19 |
| G58D | G58D | 55.621 | 0.359 | 78.716 | 2.854 |
| H74Q | H74Q | 59.562 | 0.57 | 109.633 | 13.623 |
| F108Y | F108Y | 60.063 | 0.22 | 84.862 | 5.838 |
| Y109F | Y109F | 59.547 | 0.38 | 117.431 | 1.297 |
| R113Q | R113Q | 60.019 | 0.391 | 96.789 | 3.244 |
| N130S | N130S | 59.447 | 0.371 | 98.394 | 3.568 |
| I149V | I149V | 59.766 | 0.394 | 93.578 | 7.785 |
| Q165I | Q165I | 59.905 | 0.385 | 79.817 | 7.785 |
| V174I | V174I | 61.151 | 0.403 | 90.826 | 1.297 |
| T199S | T199S | 60.957 | 0.379 | 108.716 | 5.838 |
| I204V | I204V | 59.934 | 0.638 | 105.505 | 2.595 |
| I240K | I240K | 59.119 | 0.396 | 25.046 | 0.908 |
| T251C | T251C | 60.665 | 0.467 | 102.294 | 1.946 |
| E349V | E349V | 59.937 | 0.437 | 104.128 | 8.433 |
| K351E | K351E | 59.1 | 0.473 | 135.321 | 3.244 |
| G383T | G383T | 59.556 | 0.541 | 96.789 | 0.649 |
| 2-M1 | L6P_D17V | 63.155 | 0.419 | 109.633 | 12.326 |
| 2-M2 | D17V_G58D | 57.784 | 0.428 | 108.257 | 3.892 |
| 2-M3 | D17V_H74Q | 62.246 | 0.254 | 121.56 | 3.244 |
| 2-M4 | D17V_F108Y | 62.864 | 0.217 | 92.133 | 3.341 |
| 2-M5 | D17V_Y109F | 62.269 | 0.149 | 129.817 | 5.838 |
| 2-M6 | D17V_R113Q | 62.513 | 0.143 | 121.56 | 9.731 |
| 2-M7 | D17V_N130S | 62.053 | 0.234 | 105.505 | 0 |
| 2-M8 | D17V_I149V | 65.775 | 0.328 | 94.037 | 3.244 |
| 2-M9 | D17V_Q165I | 62.193 | 0.403 | 129.358 | 2.595 |
| 2-M10 | D17V_V174I | 63.203 | 0.222 | 100.917 | 7.785 |
| 2-M11 | D17V_T199S | 63.662 | 0.316 | 107.339 | 0 |
| 2-M12 | D17V_I204V | 62.204 | 0.279 | 97.5 | 3.536 |
| 2-M13 | D17V_T251C | 63.189 | 0.232 | 110.917 | 4.022 |
| 2-M14 | D17V_E349V | 61.669 | 0.247 | 86.239 | 7.785 |
| 2-M15 | D17V_K351E | 62.225 | 0.287 | 155.046 | 1.297 |
| 2-M16 | D17V_G383T | 61.935 | 0.31 | 91.284 | 7.136 |
| 2-M17 | Y109F_V174I | 61.239 | 0.623 | 89.908 | 2.595 |
| 2-M18 | R113Q_V174I | 61.29 | 0.51 | 89.45 | 3.244 |
| 2-M19 | N130S_V174I | 61.131 | 0.519 | 88.532 | 14.921 |
| 2-M20 | I149V_V174I | 61.413 | 0.68 | 61.72 | 2.238 |
| 2-M21 | Q165I_V174I | 61.791 | 0.57 | 100.183 | 3.633 |
| 2-M22 | V174I_G383T | 61.24 | 0.563 | 83.486 | 10.38 |
| 3-M1 | L6P_D17V_T199S | 65.332 | 0.437 | 120.183 | 1.297 |
| 3-M2 | D17V_H74Q_T199S | 65.059 | 0.259 | 88.532 | 8.433 |
| 3-M3 | D17V_F108Y_I149V | 62.692 | 0.263 | 86.697 | 7.136 |
| 3-M4 | D17V_F108Y_T199S | 64.647 | 0.214 | 100.459 | 3.244 |
| 3-M5 | D17V_Y109F_I149V | 61.907 | 0.297 | 106.422 | 1.297 |
| 3-M6 | D17V_Y109F_T199S | 63.435 | 0.41 | 101.835 | 1.297 |
| 3-M7 | D17V_R113Q_I149V | 62.03 | 0.372 | 97.248 | 1.297 |
| 3-M8 | D17V_N130S_I149V | 62.194 | 0.256 | 89.908 | 15.569 |
| 3-M9 | D17V_N130S_T199S | 63.811 | 0.428 | 101.376 | 0.649 |
| 3-M10 | D17V_I149V_Q165I | 62.701 | 0.417 | 111.927 | 2.595 |
| 3-M11 | D17V_I149V_V174I | 63.715 | 0.46 | 70.642 | 7.785 |
| 3-M12 | D17V_I149V_I204V | 62.67 | 0.363 | 98.165 | 3.892 |
| 3-M13 | D17V_I149V_K351E | 62.709 | 0.199 | 148.165 | 3.244 |
| 3-M14 | D17V_I149V_G383T | 62.108 | 0.24 | 78.899 | 5.19 |
| 3-M15 | D17V_Q165I_V174I | 64.231 | 0.397 | 109.174 | 0 |
| 3-M16 | D17V_Q165I_T199S | 65.731 | 0.235 | 120.642 | 5.838 |
| 3-M17 | D17V_V174I_T199S | 65.137 | 0.225 | 98.624 | 3.244 |
| 3-M18 | D17V_T199S_I240K | 65.062 | 0.276 | 103.211 | 3.244 |
| 3-M19 | D17V_T199S_T251C | 64.57 | 0.404 | 105.505 | 0 |
| 3-M20 | D17V_T199S_E349V | 63.345 | 0.488 | 104.128 | 5.838 |
| 3-M21 | D17V_T199S_K351E | 64.281 | 0.372 | 143.578 | 8.433 |
| 4-M1 | L6P_D17V_H74Q_T199S | 65.222 | 0.549 | 100 | 6.487 |
| 4-M2 | L6P_D17V_F108Y_T199S | 65.65 | 0.334 | 109.537 | 1.917 |
| 4-M3 | L6P_D17V_N130S_T199S | 63.028 | 0.437 | 102.752 | 3.472 |
| 4-M4 | L6P_D17V_I149V_T199S | 65.143 | 0.207 | 109.743 | 1.609 |
| 4-M5 | L6P_D17V_I149V_I204V | 63.789 | 0.13 | 118.106 | 7.858 |
| 4-M6 | L6P_D17V_I149V_T251C | 64.885 | 0.116 | 86.644 | 1.234 |
| 4-M7 | L6P_D17V_V174I_T199S | 66.352 | 0.108 | 105.947 | 3.188 |
| 4-M8 | L6P_D17V_T199S_T251C | 65.407 | 0.525 | 107.339 | 1.297 |
| 4-M9 | D17V_F108Y_V174I_T199S | 66.049 | 0.067 | 69.641 | 6.158 |
| 4-M10 | D17V_I149V_Q165I_V174I | 64.352 | 0.286 | 94.495 | 0 |
| 4-M11 | D17V_Q165I_V174I_T199S | 65.756 | 0.148 | 76.36 | 7.405 |
| 4-M12 | D17V_V174I_T199S_T251C | 65.894 | 0.167 | 85.7 | 7.97 |

Table S2. T_m_ values, relative activity and prediction data for mutants in the first round of prediction.

| ID | Mutation | Tm_DSF (℃) | Error of T_m_ (±) | Relative activity (%) | Error of relative activity (±) | Thermostability prediction scores |
| --- | --- | --- | --- | --- | --- | --- |
| 5-M1 | L6P_D17V_F108Y_V174I_T199S | 66.938 | 0.126 | 121.481 | 2.592 | 0.84877 |
| 5-M2 | L6P_D17V_F108Y_T199S_T251C | 66.944 | 0.108 | 85.326 | 2.284 | 0.85199 |
| 5-M3 | L6P_D17V_I149V_V174I_T199S | 66.536 | 0.284 | 117.2 | 2.351 | 0.84652 |
| 5-M4 | L6P_D17V_V174I_T199S_T251C | 67.191 | 0.331 | 103.374 | 0.709 | 0.86596 |
| 5-M5 | D17V_F108Y_V174I_T199S_T251C | 66.612 | 0.246 | 81.911 | 2.591 | 0.84716 |
| 6-M1 | L6P_D17V_F108Y_I149V_V174I_T199S | 67.668 | 0.135 | 116.885 | 0.995 | 0.89396 |
| 6-M2 | L6P_D17V_F108Y_I149V_T199S_T251C | 67.94 | 0.403 | 77.646 | 1.296 | 0.89875 |
| 6-M3 | L6P_D17V_F108Y_V174I_T199S_T251C | 68.044 | 0.229 | 100.475 | 0.087 | 0.91186 |
| 6-M4 | L6P_D17V_F108Y_V174I_T199S_K351E | 68.24 | 0.122 | 50.247 | 0.602 | 0.91382 |
| 6-M5 | L6P_D17V_F108Y_T199S_I204V_T251C | 67.616 | 0.125 | 99.246 | 0.736 | 0.88798 |
| 7-M1 | L6P_D17V_H74Q_F108Y_V174I_T199S_T251C | 67.625 | 0.428 | 95.179 | 0.472 | 0.93477 |
| 7-M2 | L6P_D17V_F108Y_Y109F_V174I_T199S_T251C | 67.359 | 0.126 | 126.189 | 2.325 | 0.93376 |
| 7-M3 | L6P_D17V_F108Y_I149V_V174I_T199S_T251C | 67.839 | 0.234 | 110.495 | 1.365 | 0.93576 |
| 7-M4 | L6P_D17V_F108Y_V174I_T199S_I204V_T251C | 67.851 | 0.465 | 103.468 | 1.962 | 0.95811 |
| 7-M5 | L6P_F108Y_I149V_V174I_T199S_I240K_T251C | 67.273 | 0.436 | 95.133 | 10.163 | 0.93321 |
| 8-M1 | L6P_D17V_H74Q_F108Y_Y109F_V174I_T199S_T251C | 67.293 | 0.119 | 122.995 | 0.102 | 0.9633 |
| 8-M2 | L6P_D17V_H74Q_F108Y_V174I_T199S_I204V_T251C | 67.668 | 0.416 | 107.281 | 0.411 | 0.96685 |
| 8-M3 | L6P_D17V_F108Y_Y109F_V174I_T199S_I204V_T251C | 67.458 | 0.521 | 125.02 | 0.539 | 0.9638 |
| 8-M4 | L6P_D17V_F108Y_I149V_V174I_T199S_I204V_T251C | 68.025 | 0.19 | 105.997 | 0.414 | 0.97886 |
| 8-M5 | L6P_D17V_F108Y_I149V_V174I_T199S_T251C_K351E | 68.166 | 0.327 | 82.658 | 2.988 | 0.98001 |
| 9-M1 | L6P_D17V_H74Q_F108Y_Y109F_V174I_T199S_I204V_T251C | 66.986 | 0.376 | 94.505 | 0.606 | 0.98833 |
| 9-M2 | L6P_D17V_F108Y_R113Q_I149V_V174I_T199S_I204V_T251C | 68.118 | 0.245 | 103.357 | 4.746 | 0.99207 |
| 9-M3 | L6P_D17V_F108Y_I149V_Q165I_V174I_T199S_I204V_T251C | 68.866 | 0.539 | 104.593 | 0.826 | 1 |
| 9-M4 | L6P_D17V_F108Y_I149V_V174I_T199S_I204V_T251C_K351E | 68.792 | 0.52 | 81.717 | 1.908 | 0.9921 |
| 9-M5 | L6P_D17V_F108Y_I149V_V174I_T199S_I204V_T251C_G383T | 67.897 | 0.436 | 108.417 | 1.553 | 0.99141 |

Table S3. T_m_ values, relative activity and prediction data for mutants in the second round of prediction.

| ID | Mutation | Tm_DSF (℃) | Error of T_m_ (±) | Relative activity (%) | Error of relative activity (±) | Thermostability prediction scores |
| --- | --- | --- | --- | --- | --- | --- |
| 12-M1 | L6P_D17V_H74Q_F108Y_R113Q_I149V_Q165I_V174I_T199S_I204V_T251C_K351E | 69.365 | 0.377 | 69.771 | 0.036 | 0.98971 |
| 12-M2 | L6P_D17V_H74Q_F108Y_R113Q_I149V_Q165I_V174I_T199S_I204V_T251C_G383T | 68.731 | 0.311 | 105.157 | 3.997 | 0.98139 |
| 12-M3 | L6P_D17V_H74Q_F108Y_R113Q_I149V_Q165I_V174I_T199S_T251C_E349V_K351E | 68.953 | 0.258 | 74.663 | 0.911 | 0.98351 |
| 12-M4 | L6P_D17V_H74Q_F108Y_R113Q_I149V_Q165I_V174I_T199S_T251C_K351E_G383T | 68.372 | 0.381 | 78.82 | 1.131 | 0.9787 |
| 12-M5 | L6P_D17V_H74Q_F108Y_I149V_Q165I_V174I_T199S_I204V_T251C_E349V_K351E | 69.29 | 0.194 | 73.963 | 0.435 | 0.98789 |
| 12-M6 | L6P_D17V_H74Q_F108Y_I149V_Q165I_V174I_T199S_I204V_T251C_K351E_G383T | 68.283 | 0.302 | 77.584 | 0.642 | 0.97824 |
| 12-M7 | L6P_D17V_F108Y_R113Q_I149V_Q165I_V174I_T199S_I204V_T251C_E349V_K351E | 69.386 | 0.35 | 75.037 | 2.552 | 0.99814 |
| 12-M8 | L6P_D17V_F108Y_R113Q_I149V_Q165I_V174I_T199S_I204V_T251C_K351E_G383T | 68.859 | 0.176 | 79.389 | 0.084 | 0.98253 |
| 13-M1 | L6P_D17V_H74Q_F108Y_R113Q_I149V_Q165I_V174I_T199S_I204V_T251C_E349V_K351E | 69.419 | 0.363 | 87.238 | 0.327 | 0.9983 |
| 13-M2 | L6P_D17V_H74Q_F108Y_R113Q_I149V_Q165I_V174I_T199S_I204V_T251C_K351E_G383T | 68.808 | 0.27 | 80.968 | 1.231 | 0.98989 |
| 13-M3 | L6P_D17V_H74Q_F108Y_R113Q_I149V_Q165I_V174I_T199S_T251C_E349V_K351E_G383T | 68.242 | 0.266 | 86.747 | 0.422 | 0.98948 |
| 13-M4 | L6P_D17V_F108Y_Y109F_R113Q_I149V_Q165I_V174I_T199S_I204V_T251C_E349V_K351E | 69.723 | 0.154 | 97.994 | 1.58 | 0.9998 |
| 13-M5 | L6P_D17V_F108Y_R113Q_I149V_Q165I_V174I_T199S_I204V_T251C_E349V_K351E_G383T | 69.132 | 0.251 | 82.861 | 0.758 | 0.99393 |
| 14-M1 | L6P_D17V_H74Q_F108Y_Y109F_R113Q_I149V_Q165I_V174I_T199S_I204V_T251C_E349V_K351E | 69.709 | 0.425 | 68.469 | 1.059 | 1 |
| 14-M2 | L6P_D17V_H74Q_F108Y_Y109F_R113Q_I149V_Q165I_V174I_T199S_I204V_T251C_K351E_G383T | 69.352 | 0.33 | 69.186 | 0.875 | 0.98964 |
| 14-M3 | L6P_D17V_H74Q_F108Y_R113Q_N130S_I149V_Q165I_V174I_T199S_I204V_T251C_E349V_K351E | 69.366 | 0.122 | 74.906 | 1.905 | 0.99402 |
| 14-M4 | L6P_D17V_H74Q_F108Y_R113Q_N130S_I149V_Q165I_V174I_T199S_I204V_T251C_K351E_G383T | 69.012 | 0.193 | 68.285 | 1.789 | 0.9851 |
| 14-M5 | L6P_D17V_H74Q_F108Y_R113Q_I149V_Q165I_V174I_T199S_I204V_I240K_T251C_E349V_K351E | 69.361 | 0.27 | 65.487 | 1.373 | 0.99119 |
| 14-M6 | L6P_D17V_H74Q_F108Y_R113Q_I149V_Q165I_V174I_T199S_I204V_I240K_T251C_K351E_G383T | 68.486 | 0.181 | 80.628 | 0.129 | 0.9809 |
| 14-M7 | L6P_D17V_H74Q_F108Y_R113Q_I149V_Q165I_V174I_T199S_I204V_T251C_E349V_K351E_G383T | 69.382 | 0.398 | 73.261 | 1.713 | 0.99532 |
| 14-M8 | L6P_D17V_F108Y_Y109F_R113Q_I149V_Q165I_V174I_T199S_I204V_T251C_E349V_K351E_G383T | 69.306 | 0.369 | 76.014 | 1.326 | 0.9853 |
| 15-M1 | L6P_D17V_H74Q_F108Y_Y109F_R113Q_I149V_Q165I_V174I_T199S_I204V_T251C_E349V_K351E_G383T | 69.229 | 0.144 | 73.779 | 1.182 | 0.99545 |
| 15-M2 | L6P_D17V_H74Q_F108Y_Y109F_N130S_I149V_Q165I_V174I_T199S_I204V_I240K_T251C_E349V_K351E | 69.645 | 0.102 | 85.361 | 1.162 | 0.9852 |
| 15-M3 | L6P_D17V_H74Q_F108Y_R113Q_N130S_I149V_Q165I_V174I_T199S_I204V_T251C_E349V_K351E_G383T | 68.973 | 0.219 | 78.698 | 1.01 | 0.99141 |
| 15-M4 | L6P_D17V_F108Y_Y109F_R113Q_N130S_I149V_Q165I_V174I_T199S_I204V_I240K_T251C_E349V_K351E | 69.245 | 0.085 | 87.505 | 0.81 | 0.98656 |


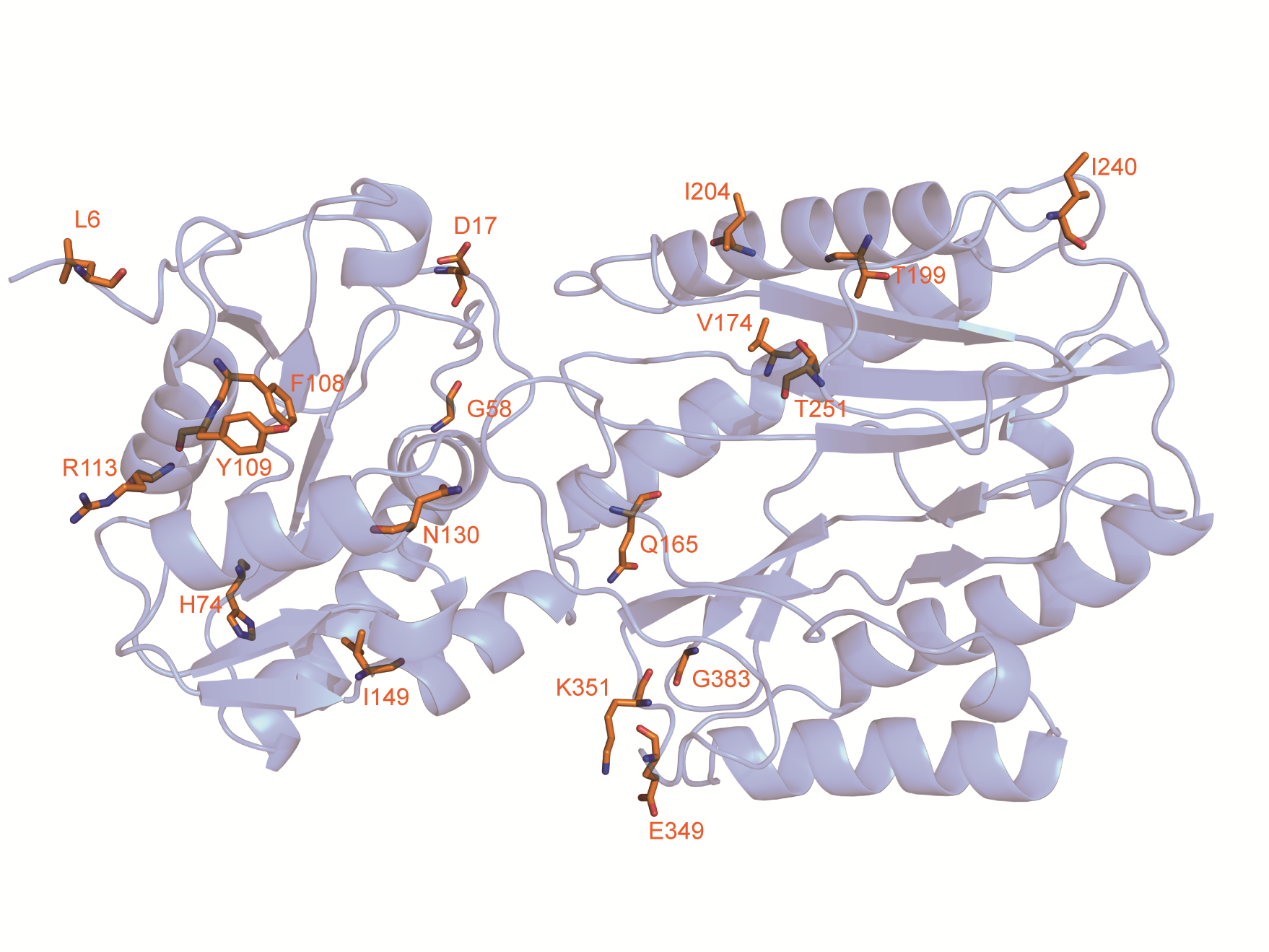


Figure S1. Distribution of the single-point mutations in the creatinase structure. Amino acid structures displayed as stick representations with amino acid types and names labeled in orange font.

**
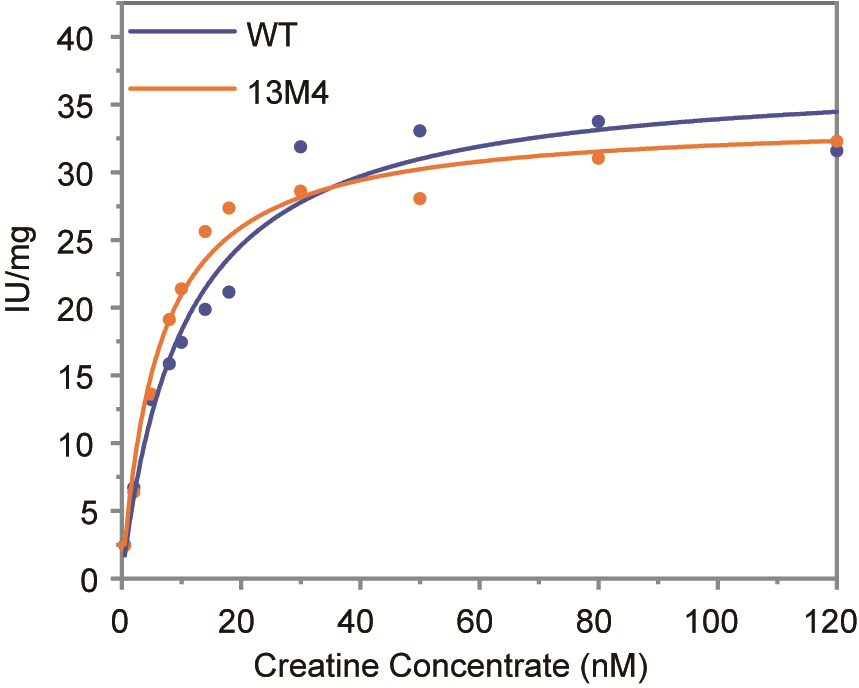
**

Figure S2. Catalytic kinetics of WT and 13M4. The kinetic parameters were calculated by fitting the Michaelis-Menten equation using Origin Pro software.


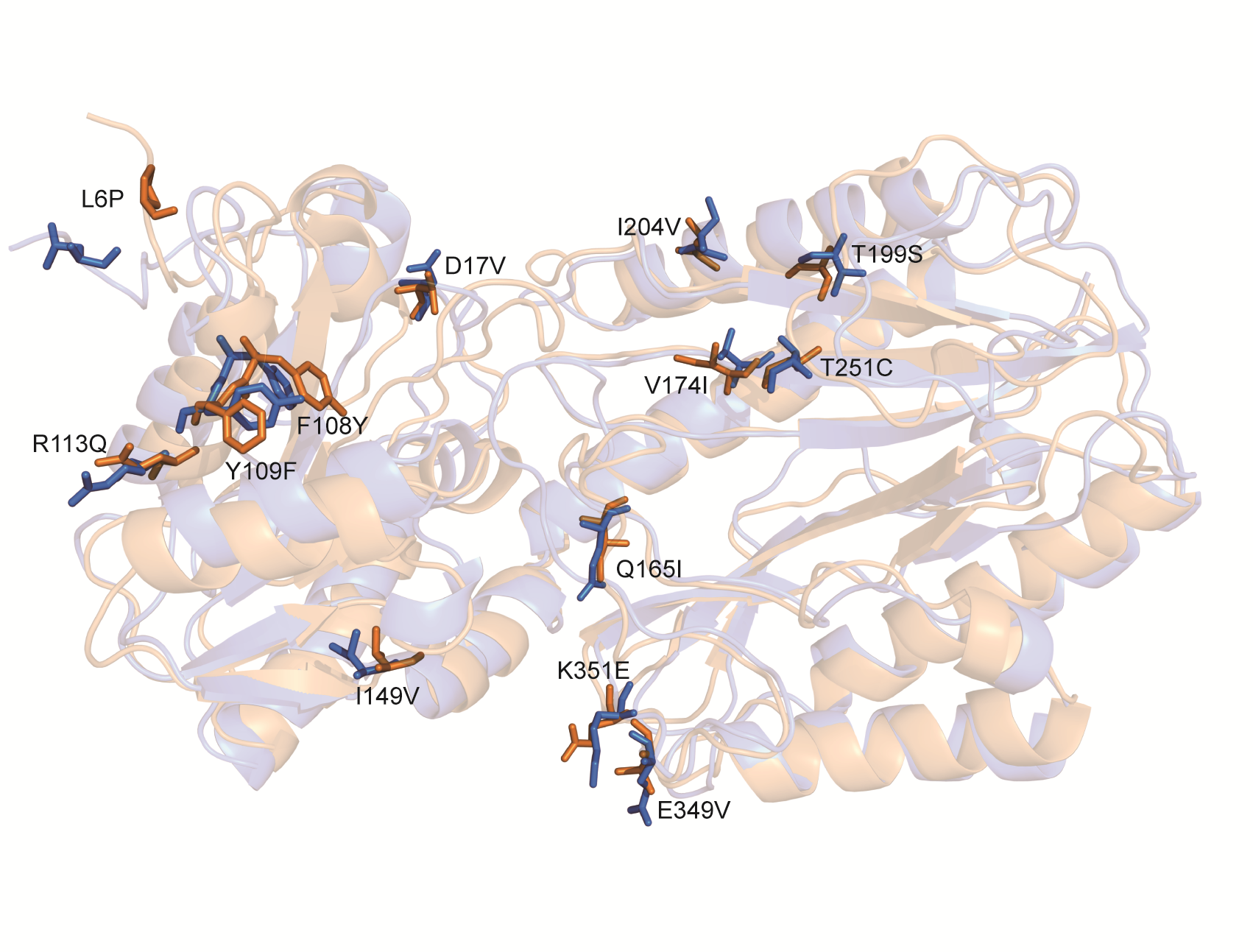


Figure S3. Distribution of the mutated amino acids in the structures of WT and 13M4.The WT structure is shown in blue cartoon, while the 13M4 structure is shown in orange cartoon. The amino acids before and after the mutation are represented by blue and orange ball-and-stick structures, respectively. The names and positions of the amino acids are labeled in black text.


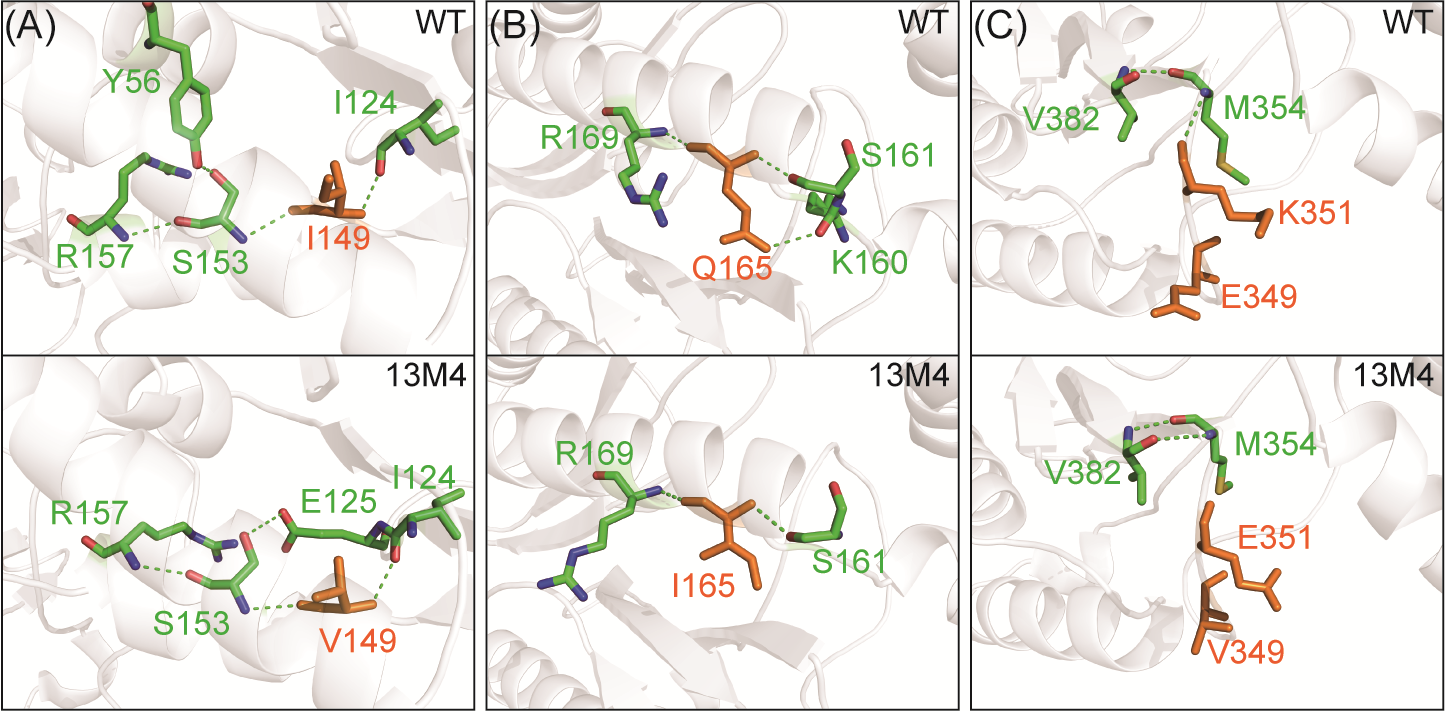


Figure S4. Structural comparison between WT and 13M4 near the mutations. Specifically, (A)-(C) represent the inter-residue interactions around the mutation respectively: I149V, Q165I and E349V /K351E. The mutated amino acids are shown in orange. Residues involved in hydrogen bonding and van der Waals interactions are shown in green, and gray, respectively. Hydrogen bonds are shown as green dashed lines.


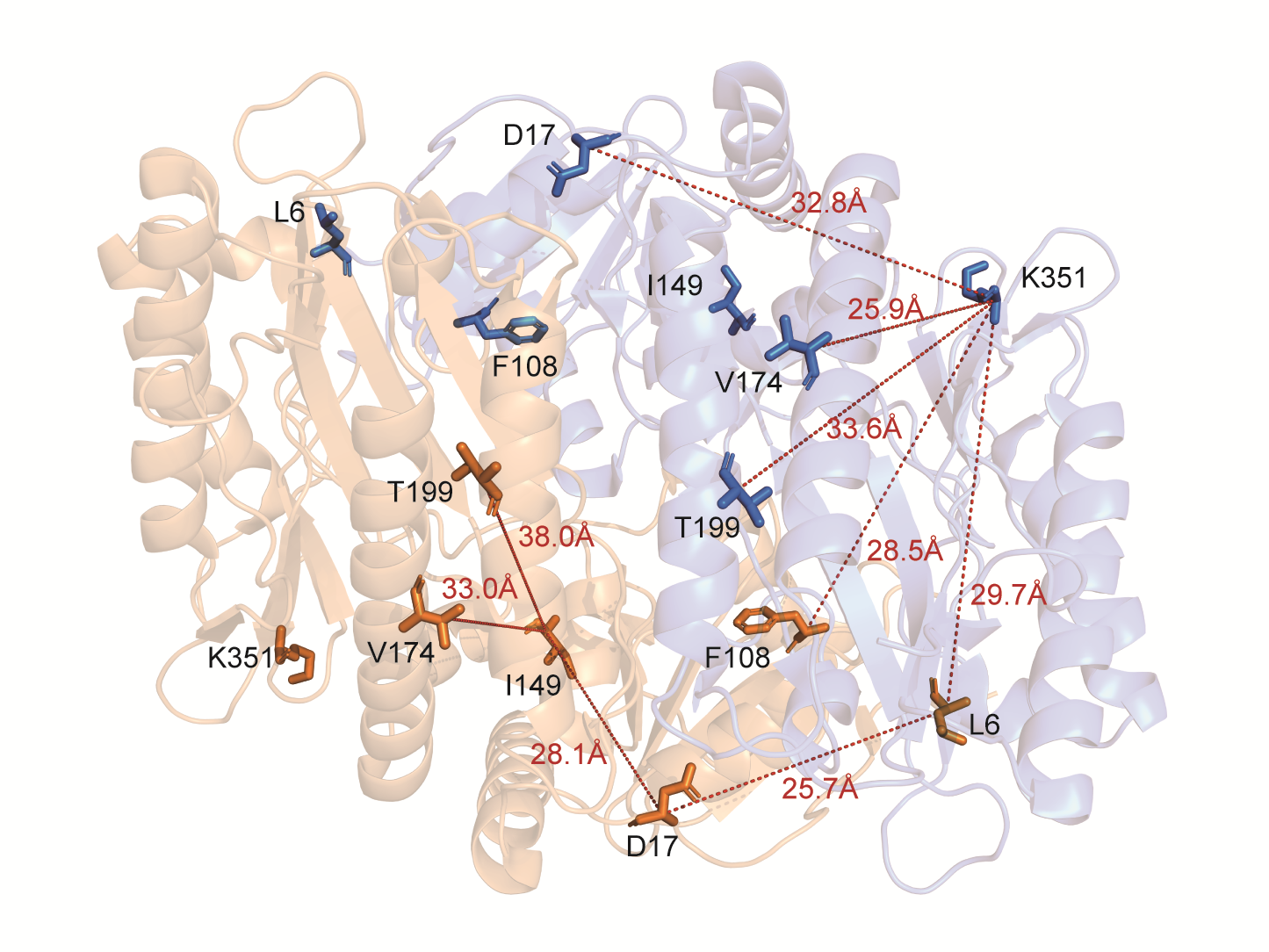


Figure S5. Distances between the mutations with epistasis. The creatinase dimeric structure is depicted with the two chains shown in blue and orange cartoon representations. Distances between the mutated amino acids are delineated by red dashed lines, with distance values annotated in red.
